# Supplementary material for: GhFAD3-4 Promotes Fiber Cell Elongation and Cell Wall Thickness by Increasing PI and IP3 Accumulation in Cotton
Source: Plants (Basel). 2024 May 30;13(11):1510. doi: 10.3390/plants13111510 (PMC11174750; doi:10.3390/plants13111510)
Supplement: Supplementary file 1 [file plants-13-01510-s001.zip › plants-2985854-supplementary.pdf]

GhFAD3-4 promotes fiber cell elongation and cell wall thickness by increasing PI and IP_3 accumulation in cotton

Huiqin Wang[1], Mengyuan Fan[1], Yongcui Shen[1], Hanxuan Zhao[1], Shuangshuang Weng, Zhen Chen[1], Guanghui Xiao[1,*]

[1]College of Life Sciences, Shaanxi Normal University, Xi'an 710062, China.

*Correspondence：Guanghui Xiao E-mail: guanghuix@snnu.edu.cn

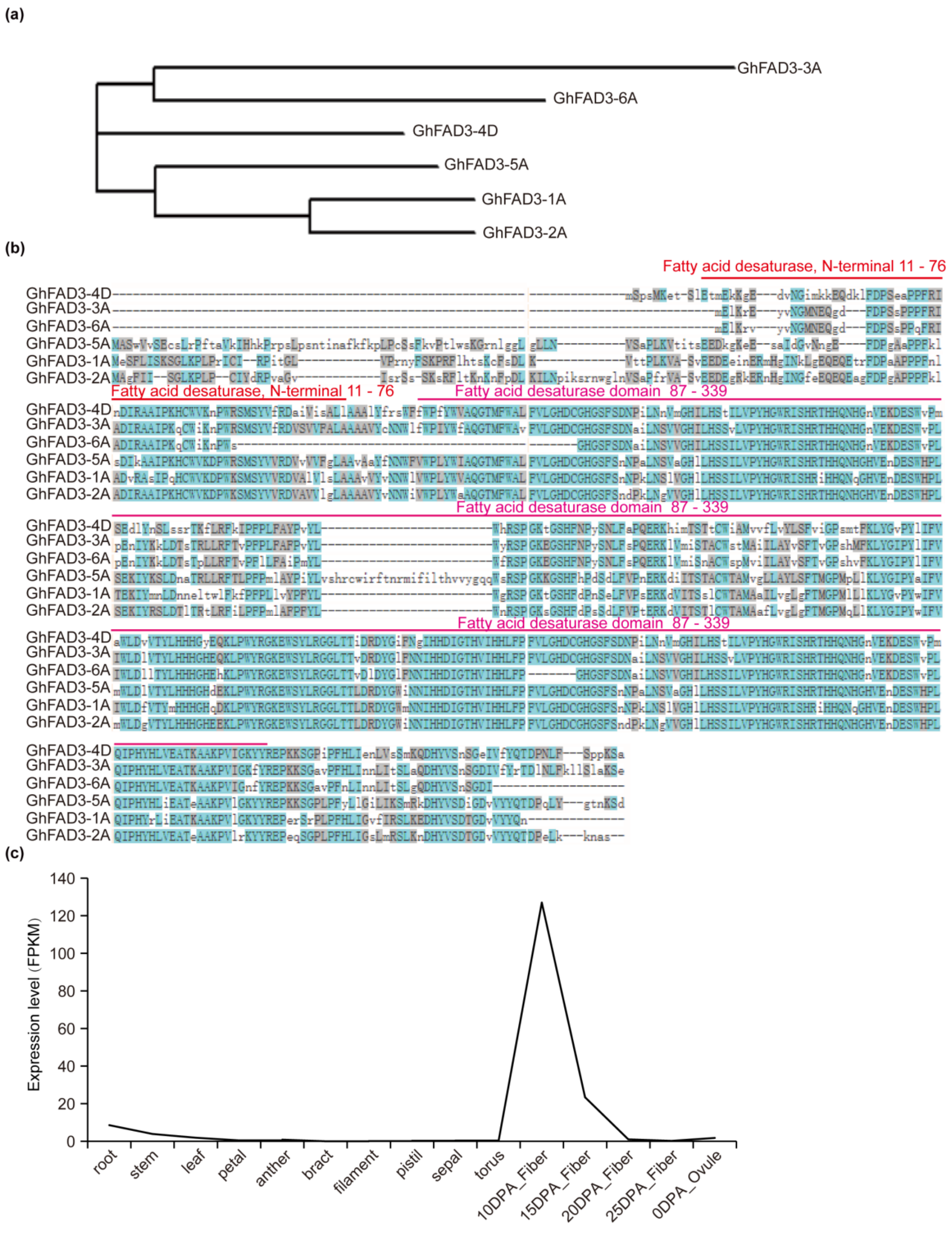

Figure S1. Phylogenetic tree and sequence alignment of *GhFAD3-4* homologs in cotton.

(a) Phylogenetic tree of *GhFAD3-4* homologs in cotton.

(b) Sequence alignment of *GhFAD3-4* homologs in cotton.

(c) Transcriptome analysis of *GhFAD3-4* in cotton.

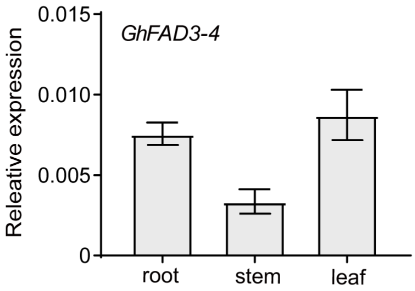

Figure S2. Analysis of *GhFAD3-4* expression levels in cotton roots, stems and leaves.

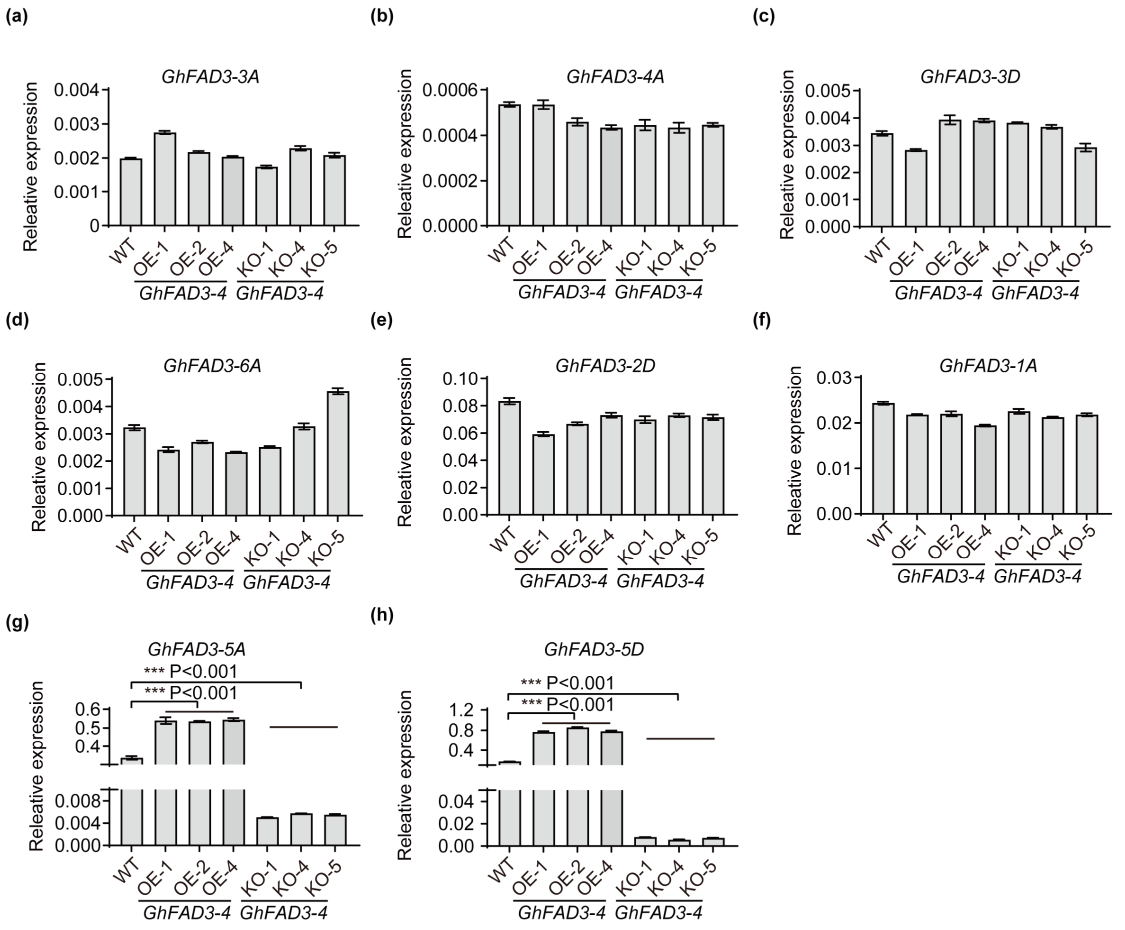

Figure S3. Expression analysis of *GhFAD3-4* homologs in *GhFAD3-4* transgenic lines.

(a) -(h) The expression levels of *GhFAD3-4* homologs (*GhFAD3-1A*, *GhFAD3-2D*, *GhFAD3-3A*, *GhFAD3-4A*, *GhFAD3-4D*, *GhFAD3-5A*, *GhFAD3-5D*, *GhFAD3-6A*) in *GhFAD3-4* transgenic materials.

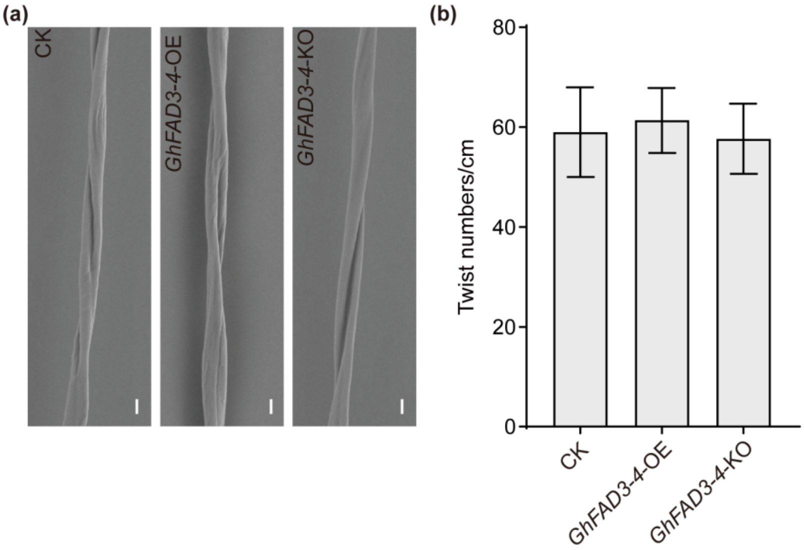

Figure S4. Fiber twist analysis of *GhFAD3-4* transgenic materials.

(a) Fiber twist phenotype of *GhFAD3-4* transgenic lines. Scale bar = 10 μm.

(b) Statistical results of **(a)**.

Table S1 The list of primers used in the study

**Identification and construction of transgenic materials**	
35S-F	CAATCCCACTATCCTTCGCAAGACC
GhFAD3-4-OE-R	TCATGCGGATTTGGGAGGA
GhFAD3-4-OE-vector-F	GAGAACACGGGGGACGGATCCATGAGCCCTTCAATGAAGGAAACA
GhFAD3-4-OE-vector-R	ACCGTCATGGTCTTTGTAGTCCATTGCGGATTTGGGAGGAGAAAA
GhFAD3-4-Cas9-F	CCCTTGGAGGTCTATGAGTTATGTTT
GhFAD3-4-Cas9-R	GGCCTTTCTACTGGGTTGCCCAAGGG
**gRNA sequence**	
GhFAD3-4T1	CATAACTCATAGACCTCCAA
GhFAD3-4T2	CTTTCTACTGGGTTGCCCAA
**qRT-PCR**	
QRT-GhBoGH3B-F	GTCTAGGTGCAAGCAGGCAT
QRT-GhBoGH3B-R	CACCGCTATACAAGGTGCGA
QRT-GhMIOX4-F	GGAGAAGCCTGAGCTAGTGTC
QRT-GhMIOX4-R	CCACGCTCTTTTGCCTTTCA
QRT-GhRNF144B-F	TAACAGGTGGGGAAAAGCCC
QRT-GhRNF144B-R	TGCACTGCACGCAAAATACC
QRT-GhFAD3-1A-F	CGCCATTGAAAGTTGCATCCGT
QRT-GhFAD3-1A-R	GGGAGCTGCAGGGTCAAATCTT
QRT-GhFAD3-2D-F	ATCATGGCACCCGTTGTCTGAG
QRT-GhFAD3-2D-R	GGTCGAAGTGCGAACCACTCTT
QRT-GhFAD3-3A-F	CATGGGTTCCGTTGCCTGAGAA
QRT-GhFAD3-3A-R	ACACGGGGAATGCAAATAGGGG
QRT-GhFAD3-3D-F	GAAACTCGACACCAGTACGCGA
QRT-GhFAD3-3D-R	CTTTGCCTGGGCTTCTATGCCA
QRT-GhFAD3-4A-F	GGGACCATGTTTTGGGCTGTCT
QRT-GhFAD3-4A-R	CGGACATCGGAACCCAAGACTC
QRT-GhFAD3-5A-F	GAAGCTACCGAAGCAGCAAAGC

QRT-GhFAD3-5A-R	ACACGACATCACCAATGTCACTCA
QRT-GhFAD3-5D-F	GAAGCTACCGAAGCAGCAAAGC
QRT-GhFAD3-5D-R	ACACGACATCACCAATGTCACTCA
QRT-GhFAD3-6A-F	CACCAGTACGCCATTACTGCGA
QRT-GhFAD3-6A-R	AACACGTGGGATGGACCAACTG
